# Identification of 613 new loci associated with heel bone mineral density and a polygenic risk score for bone mineral density, osteoporosis and fracture

**DOI:** 10.1371/journal.pone.0200785

**Published:** 2018-07-26

**Authors:** Stuart K. Kim

**Affiliations:** Department of Developmental Biology, Stanford University Medical Center, Stanford, California, United States of America; Van Andel Institute, UNITED STATES

## Abstract

Low bone mineral density (BMD) leads to osteoporosis, and is a risk factor for bone fractures, including stress fractures. Using data from UK Biobank, a genome-wide association study identified 1,362 independent SNPs that clustered into 899 loci of which 613 are new. These data were used to train a genetic algorithm using 22,886 SNPs as predictors and showing a correlation with heel bone mineral density of 0.415. Combining this genetic algorithm with height, weight, age and sex resulted in a correlation with heel bone mineral density of 0.496. Individuals with low scores (2.2% of total) showed a change in BMD of -1.16 T-score units, an increase in risk for osteoporosis of 17.4 fold and an increase in risk for fracture of 1.87 fold. Genetic predictors could assist in the identification of individuals at risk for osteoporosis or fractures.

## Introduction

Low bone mineral density (BMD) is the major determinant for osteopenia and osteoporosis, which increase the chances of fragility fracture especially for elderly women [1,2]. Low BMD is also a risk factor for bone fractures, especially stress fracture [3,4]. For athletes and military personnel undergoing harsh rigors of training, stress fractures, are common injuries that limit playing time, military effectiveness and competitive success [3,5,6]. BMD can be assessed in the lumbar spine or femoral neck by dual-energy X-ray absorptiometry (DXA), which is a non-invasive method to assess BMD that is not always widely available. Quantitative ultrasound of the calcaneus is a second method to measure BMD (estimated heel BMD, or eBMD). Quantitative ultrasound is mobile, inexpensive, easy to perform and radiation-free, and can predict fractures to the same extent as DXA.

Twin- and family-based studies have found that heel BMD has a strong genetic component with estimates of heritability of 0.45 to 0.82 [7–11]. Previous studies have identified DNA variants with genetic associations with BMD. Estrada et al. performed a meta-analysis of 17 genome-wide association studies for bone mineral density of the femoral neck or the lumbar spine, leading to the identification of 62 SNPs showing genome-wide significant associations [12]. Maoyyeri et al. and Mullin et al. performed meta-analyses for genetic association with heel BMD, and identified 9 and 13 SNPs with genome-wide significant associations, respectively [13,14]. Finally, Kemp et al. performed a genome-wide association screen for association with eBMD using data from an intermediate release from UK Biobank [15]. They identified 307 independent SNPs with genome-wide significant associations to heel BMD that were clustered in 203 loci.

In this study, a genetic association analysis for eBMD was performed on the full release of data from the UK Biobank. The analysis identified SNPs with independent genome-wide significant associations with eBMD and also generated a risk score for low BMD, osteoporosis and risk of fracture. Knowledge of one’s genetic risk for low BMD could be combined with clinical risk factors to identify patients, athletes or military personnel at high risk for osteoporosis and fracture, thereby permitting treatment or training protocols to reduce their risk [16,17].

## Results

### Identification of DNA variants associated with eBMD

Phenotypic data on eBMD, height, weight, age and sex were obtained from UK Biobank (Methods)(URLs). Genotypic data for each participant were obtained from UK Biobank [18]. Participants were restricted to those with European ancestry to reduce population stratification, and then further filtered as described in Methods. The result was a cohort of 394,929 individuals with genotype and phenotype data (Table 1). The DNA variants were restricted to those with a minor allele frequency > 0.001, resulting in a set of 20,259,828 genotyped and imputed DNA variants from the autosomes and the X chromosome, 1,270 genotyped DNA variants from the pseudo-autosomal region of the Y chromosome, 265 mitochondrial DNA variants and 362 HLA loci. The DNA variants in each of these regions of the genome were analyzed for genetic associations with eBMD.

**Table 1:** Demographic characteristics of the UK Biobank cohort.

Sex		Females	Males
		(N = 217,674; 55%)	(N = 177,253; 45%)
	Unit	Mean (95% CI)	Mean (95% CI)
Age at ascertainment	yrs.	56.6 (41.0 to 72.2)	57.0 (41.1 to 72.9)
Height	cm.	162.7 (150.5 to 174.9)	175.9 (162.6 to 189.1)
Weight	kg.	71.4 (44.3 to 98.5)	85.8 (58.6 to 113.0)
eBMD	T-score	-0.61 (-2.59 to 1.37)	-0.14 (-2.29 to 2.00)

**Fig 1:**
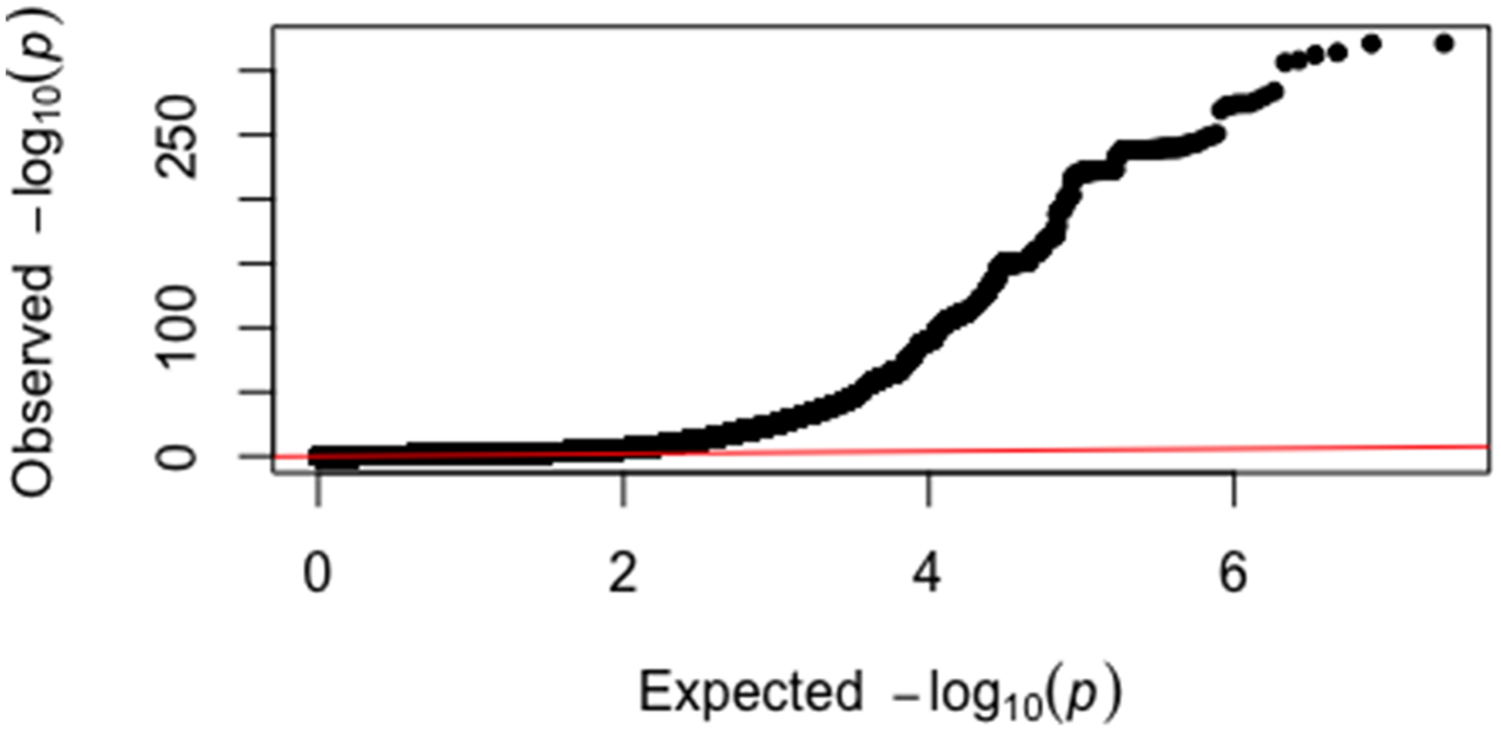
Quantile-quantile plot for genome-wide association analysis of eBMD. The expected versus observed log transformed values for the 20,259,828 p-values from the LMM analysis are graphed. The y-axis shows the observed p-values and the x-axis shows the p-values expected by chance. The black dots represent the SNPs arranged by their observed p-values and the red line shows the expected trajectory if the SNPs had p-values expected by chance.

For the X and autosomal chromosomes, genetic association was determined using a linear mixed model in order to account for population structure in the cohort (i.e. presence of related individuals) using LMM-BOLT (Methods)[19]. The model included array batch, sex, age, height, weight and the leading 10 genomic principal components as covariates. A Q-Q plot of all tested DNA variants shows a large deviation from a random distribution, indicating that many variants are likely to have an association with eBMD (Fig 1). There was substantial inflation of the test statistics relative to the null (**λ**_GC_ = 1.59) and the linkage disequilibrium score regression intercept was 1.21. These results indicate that the majority of inflation is due to polygenicity rather than population stratification.

**Fig 2:**
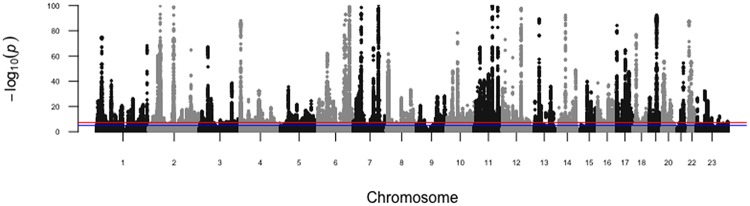
Manhattan plot for genome-wide association analysis of eBMD. The -log_10_ p-values for association with eBMD of SNPs from the LMM analysis are plotted by genomic position with chromosome number listed across the bottom. The y-axis shows the -log_10_ p-value for association with eBMD. The blue line represents suggestive genome-wide significance (p<5x10^-5^) and the red line represents genome-wide significance (p<6.6x10^-9^).

In total, 142,417 DNA variants from the autosomes and X chromosome were associated with eBMD with genome-wide significance (p<6.6x10^-9^)(S1 Table). For DNA variants in the HLA region, the Y chromosome and the mitochondria, genetic associations were analyzed by linear regression using PLINK2.0 [20]. The linear regression model included height, age, sex and weight as covariates. None of the DNA variants from the HLA loci, the Y chromosome or the mitochondria showed genome-wide significant associations with eBMD [20]. Fig 2 shows a Manhattan plot of the tested SNPs.

Conditional and joint association analysis was used to identify 1,362 DNA variants showing an independent genome-wide significant association with BMD, using GCTA COJO [21](S2 Table). Most of the independent DNA variants were spaced widely apart from each other, but some were clustered together suggesting that they were alleles affecting the same genetic locus, a phenomenon known as allelic heterogeneity. DNA variants were assigned to the same locus if they were less than 100 kb apart, resulting in 899 genetic loci.

**Fig 3:**
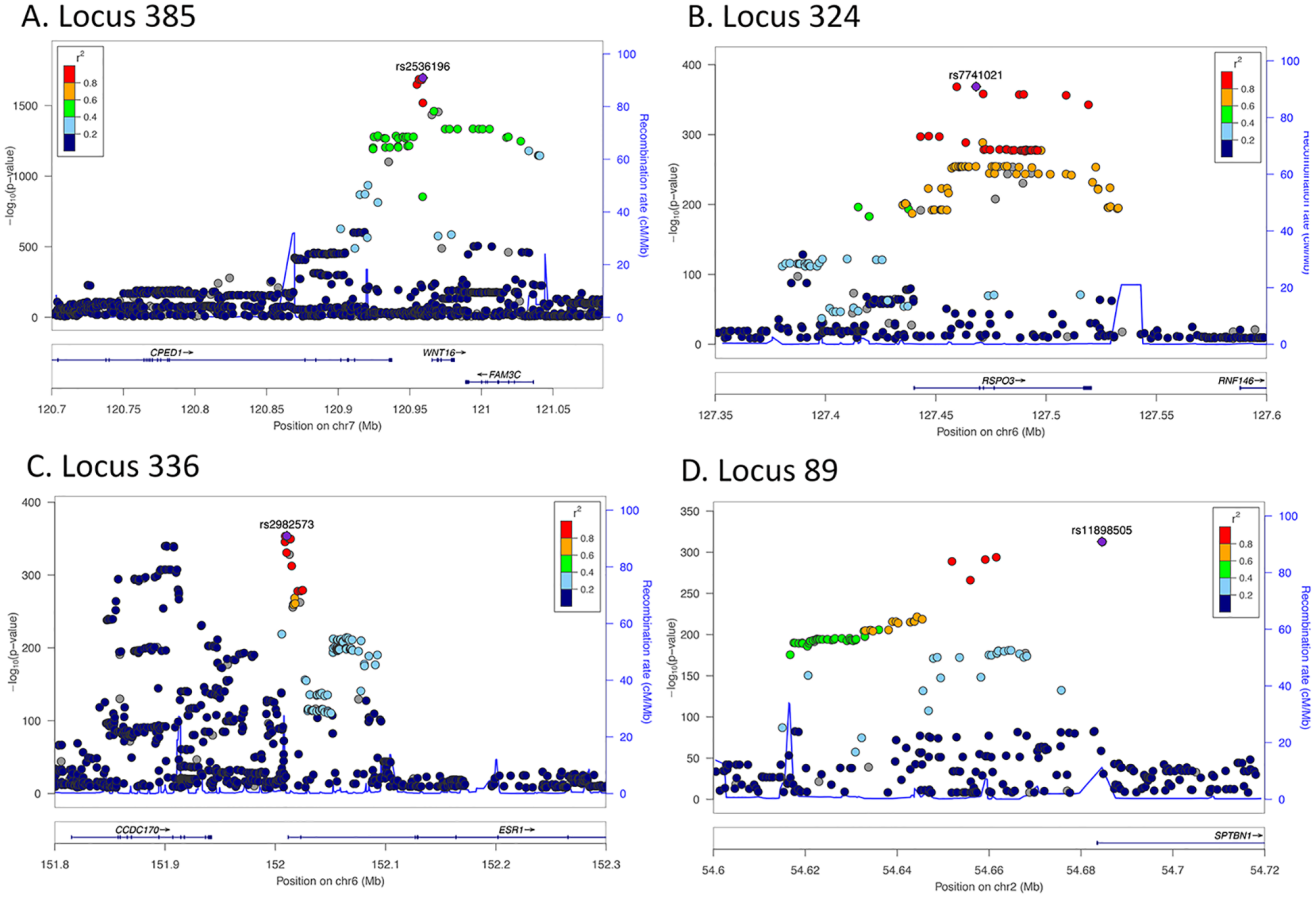
Regional-association plots for eBMD. A. Locus 385. B. Locus 324. C. Locus 336. D. Locus 89. Tested SNPs are arranged by genomic position (x-axis) around the lead SNP (purple diamond). The y-axis on the left indicates -log_10_ p-values for association with eBMD for each SNP. The y-axis on the right indicates recombination rate shown with a blue line. The color of dots of the flanking SNPs indicates their linkage disequilibrium (R^2^) with the lead SNP as indicated by the heat map color key.

Fig 3 shows the four loci with the strongest associations with eBMD: loci 385, 324, 336 and 89. Locus 385 on chromosome 7 contains 20 independent SNPs with genome-wide significant associations with eBMD. The sentinel SNP (rs2536196; p = 2.2x10^-1653^) lies within the *WNT16* gene (encodes a WNT signal). Locus 324 is on chromosome 6 and contains six independent genome-wide significant SNPs. The SNP with the strongest association to eBMD (rs9482773; p = 2.6x10^-369^) is located within the *RSPO3* gene (encodes R-spondin 3). Locus 336 is on chromosome 6 and contains 13 independent SNPs with genome-wide associations with eBMD. The sentinel SNP (rs2982573; p = 1.9x10^-354^) is located between the *CCDC170* gene (encodes Coiled-Coil Domain-Containing protein 170) and the *ESR1* gene (encodes Estrogen receptor alpha). Locus 89 on chromosome 2 contains 4 independent SNPs, with the strongest SNP (rs11898505; p = 1.4x10^-313^) lying within the *SPTBN* gene (encodes Beta spectrin non-erythrocytic protein 1).

**Fig 4:**
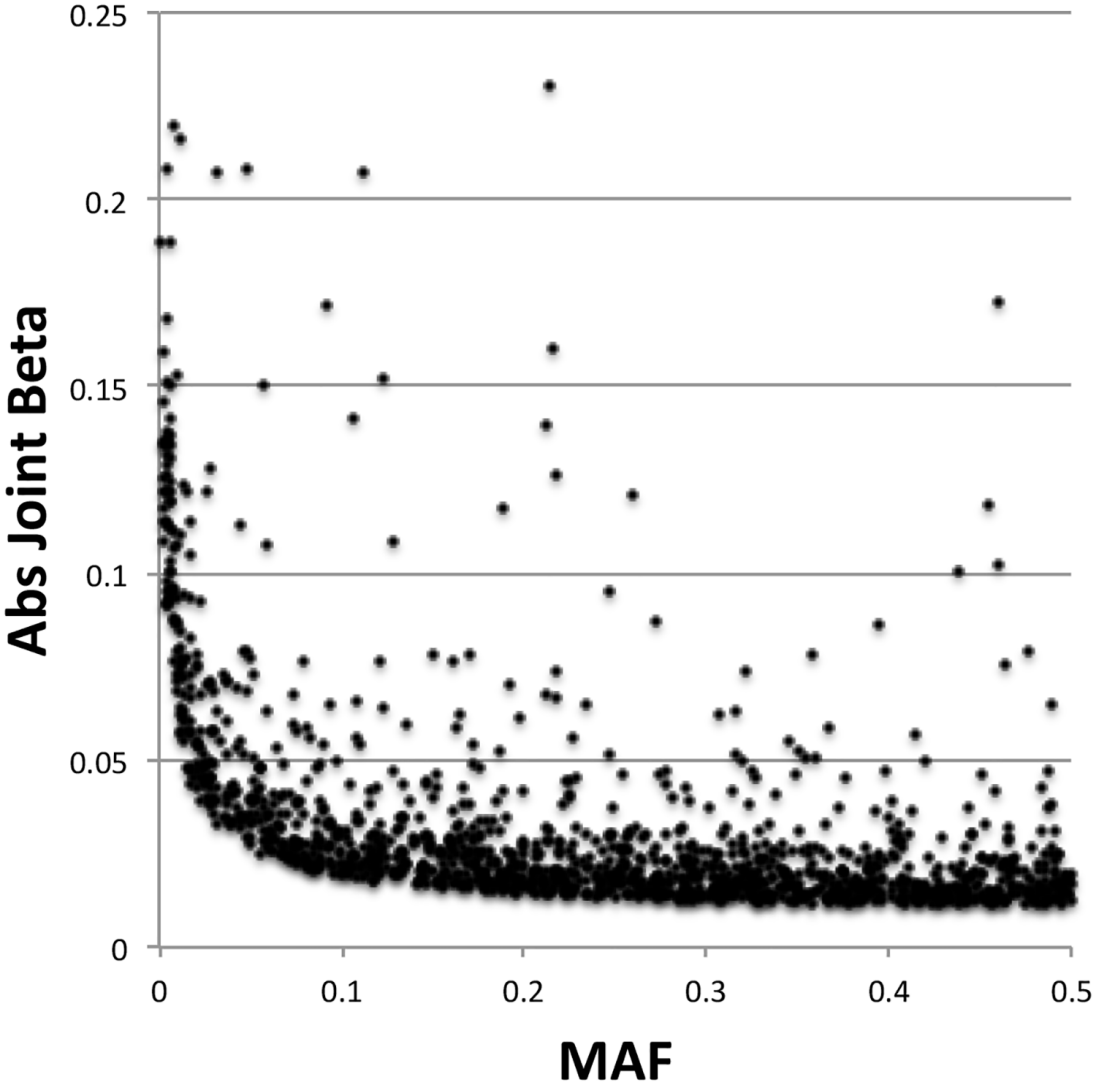
Relationship between effect size and minor allele frequency. Shown is a scatterplot of 1362 SNPs with an independent association with eBMD. The x-axis is the minor allele frequency. The y-axis is the absolute value of the beta coefficient from the joint and conditional analysis.

Since negative selection of alleles with deleterious effects would tend to lower their allele frequency, the relationship between effect size and minor allele frequency was examined for the 1362 SNPs with independent associations with eBMD (Fig 4). There was a trend for alleles with low frequency to have stronger effect sizes, as had been observed previously by Kemp et al. [15].

### Candidate SNPs that may play a causal role in altering eBMD

To find candidate causal variants within each locus, the set of 142,417 genome-wide significant SNPs were filtered to find DNA variants resulting in changes in either protein sequence or expression level. First, 581 DNA variants were identified resulting in alterations to protein sequence using Annovar (Methods)[22]. From these, 52 were identified that were the sentinel SNP for their locus and 393 had a p-value comparable to the sentinel SNP (Methods)(S3 Table). These 445 SNPs are candidates for being responsible for genetic association with eBMD via alteration of the function of a specific protein.

Second, 2,124 SNPs were identified as candidates for mutations leading to changes in expression of a gene involved in setting eBMD using regulomeDB (S4 Table; Methods)[23]. These SNPs were located within a transcription factor binding region, as defined by Chromatin Immunoprecipitation-Sequencing experiments performed by the ENCODE consortium [24]. The SNPs were also associated with changes in expression of a nearby gene, referred to as cis Expression Quantitative Trait Loci (cis-eQTLs). DNA variation within a transcription factor binding site could lead to changes in binding of that transcription factor, which could in turn result in altered expression of a nearby gene, which could affect eBMD if the gene is involved in bone density.

Although the 445 protein-altering SNPs and the 2,124 regulatory SNPs are good candidates for being causal mutations for altering eBMD, it should be noted that these DNA variants might instead be passive bystanders that are correlated with eBMD but do not causally change it. For instance, there might be a rare SNP that is currently undiscovered and tightly linked to one of these candidate SNPs that is the true causal mutation; an example of this can be seen in SNPs associated with human height where protein coding SNPs are linked to regulatory SNPs [25].

### Validation of previous associations with eBMD

The results from this study were compared to previous genome-wide association studies of BMD. Estrada et al. performed a meta-analysis for genetic association to bone mineral density of the femoral neck or the lumbar spine, and identified 62 SNPs showing genome-wide significant associations [12]. Of these, 57 SNPs were replicated in the UK Biobank data as they had beta values in the same direction and p-values below p = 8x10^-4^, which is the cutoff for Bonferroni significance (S5 Table). Maoyyeri et al. performed a meta-analysis for genetic association with eBMD, and identified nine SNPs with genome-wide significant associations [13]. All nine of the previously-identified SNPs were strongly replicated using the UK Biobank data, with beta values in the same direction and p≤5.9x10^-71^ in every case (S5 Table). Mullin et al. identified 13 SNPs with genome-wide significant associations to eBMD [14]. Of these, all 13 were validated in this study; in 12 cases, the identical SNP was validated and in one case, the specific SNP was missing but a nearby proxy SNP showed an association (S5 Table). Finally, Kemp et al. performed a genome-wide association screen for association with eBMD using data from an intermediate release from UK Biobank (142,487 individuals)[15]. Their study identified 307 independent SNPs with genome-wide significant associations to eBMD located in 203 loci. Since half of the data from this current study overlap the data from Kemp et al., the data from this study are not an independent test but can be used to support the general trends from Kemp et al. Using data from the current study, 274 of the 307 previously-identified SNPs had beta values in the same direction and p-values below 1.6x10^-4^ (i.e. Bonferroni cutoff) for association with eBMD (S5 Table). For another 30 of the previously-identified SNPs, the specific SNP was not included in the present study but there was a SNP within 100 kb that had a significant p-value and beta values in the same direction. In total, 304 of the 307 SNPs previously-identified by Kemp et al. were supported by the data in this current study, either using the direct SNP itself or a proxy SNP. Of the 899 loci identified in this work, 286 were identified in one of the previous three studies and 613 are new.

### Genetic association with bone fracture

Since low bone mineral density is a risk factor for bone fracture [26], a genetic association analysis was performed for incidence of fracture. There were 59,378 participants in the UK Biobank that had any type of bone fracture and 348,296 controls; the cases of fracture were identified by self-reported responses to a questionnaire (47,494 individuals) or by inpatient electronic health records (23,686 individuals)(S6 Table). Linear mixed modeling was used to assess genetic association with bone fracture using the same covariates as was used for eBMD (array batch, height, weight, age, sex and the first 10 principal components)(Methods). Of the 142,417 SNPs showing a genome-wide significant association to eBMD, 1,523 SNPs (1.1%) showed a significant association to bone fracture (using p = 3.56x10^-7^ as a cutoff for Bonferroni significance)(S1 Table).

### Polygenic risk score for bone mineral density, osteoporosis and bone fracture

The genetic association data were used to construct risk scores to predict bone mineral density, risk for osteoporosis or risk for fracture. Three methods were tried: 1) a polygenic risk score (PRS) using the beta values from genome-wide significant SNPs, similar to the method used in Estrada et al. [12], 2) a Bayesian approach that estimates effect sizes for each variant based on its summary statistics for association with eBMD (LDpred) and 3) a least absolute shrinkage and selection operator method (LASSO) to select predictors and calculate their weights.

In the first method, the cohort was split into a training set (350,000 individuals) and a validation set (44,924 individuals). Linear mixed modeling was used to identify 121,536 SNPs with genome-wide significant associations with eBMD (i.e. p<6.6x10^-9^). From these SNPs, conditional and joint association analysis was used to identify 1,182 SNPs with independent genome-wide significant associations to eBMD (Methods). A polygenic risk score (termed the GWS_PRS) was created where the score is the sum of the number of effect alleles weighted by the beta coefficient for each of the 1,182 independent SNPs (Table 2).

**Table 2:** Performance of polygenic risk scores for eBMD.

Name		Predictors^a^	Covariates	Correlation BMD^b^ (95% CI)
GWS_PRS	NA	1,182	None	0.370 (0.362 to 0.378)
	Fraction Causal			
LDpred1	1.0	1,497,447	None	0.305 (0.296 to 0.313)
LDpred2	0.3	1,497,447	None	0.231 (0.222 to 0.240)
LDpred3	0.1	1,497,447	None	0.219 (0.210 to 0.227)
LDpred4	0.03	1,497,447	None	0.238 (0.230 to 0.247)
LDpred5	0.01	1,497,447	None	0.201 (0.192–0.210)
LDpred6	0.003	1,497,447	None	0.143 (0.134 to 0.152)
LDpred7	0.001	1,497,447	None	0.137 (0.127 to 0.146)
LDpred8	0.0003	1,497,447	None	0.084 (0.075 to 0.093)
LDpred9	0.0001	1,497,447	None	0.086 (0.077 to 0.095)
	Lambda^c^			
Lasso1	1X10^-2^	1,306	None	0.354 (0.346 to 0.362)
Lasso2	3X10^-3^	6,204	None	0.408 (0.401 to 0.416)
Lasso3	2X10^-3^	11,452	None	0.413 (0.405 to 0.421)
Lasso4	1X10^-3^	22,886	None	0.415 (0.407 to 0.422)
Lasso5	6X10^-4^	32,012	None	0.412 (0.404 to 0.420)
Lasso6	3X10^-4^	45,683	None	0.402 (0.394 to 0.410)
Lasso7	1x10^-4^	71,845	None	0.373 (0.365 to 0.381)
Lasso8	1x10^-5^	131,004	None	0.320 (0.311 to 0.328)
Estrada_GRS^d^	NA	63	None	-0.143 (-0.154 to -0.132_
Covar^e^	NA	NA	Ht, Age, Wt, Sex	0.251 (0.240 to 0.262)
BOG^f^	1x10^-3^	22,886	Ht, Age, Wt, Sex	0.496 (0.489 to 0.503)

^a^ Number of predictors used in model.

^b^ Correlation of PRS with eBMD in cohort C.

^c^ lambda penalty value used in LASSO.

^d^ 63 SNPs used in Estrada et al., 2012 [12].

^e^ Risk score based on height, weight, age and sex.

^f^ BMD Osteoporosis Genetic risk score based on LASSO4, height, weight, age and sex.

For generating genetic risk scores for highly polygenic traits, prior studies have shown that restricting predictor SNPs to those that have genome-wide significance is too restrictive, as many SNPs with weaker p-values are still informative [21,27]. Regression analysis can be used to select SNPs with p-values below the genome-wide significant cutoff, where the information from the aggregate set of SNPs is informative even though the reliability of each individual SNP is less than that of genome-wide significant SNPs. The second (LDpred) and third (LASSO) methods both use a large number of predictor SNPs, including those with p-values below genome-wide significance, in order to generate a PRS for bone mineral density [19,28].

LDpred uses a Bayesian approach that estimates effect sizes for each variant based on its summary statistics for association with eBMD, as modified by linkage disequilibrium between it and other SNPs from a reference panel (Methods)[19]. The summary statistics were generated using linear mixed modeling from 350,000 participants, and then validated on the remaining 44,924 individuals. The PRS generated by LDpred is dependent on a variable indicating the fraction of SNPs showing a non-zero association with BMD. This variable is not known, and so it was set to one of nine values between 10^−4^ and 1. This resulted in the generation of seven PRS’s for eBMD, each containing effect sizes for 1,497,447 SNPs (Table 2).

The third training method used the least absolute shrinkage and selection operator (LASSO) to select predictors and calculate their weights, using PLINK1.9 [20]. For this method, the UK Biobank cohort was divided into three parts: discovery of candidate SNPs (cohort A), training genetic algorithms (cohort B) and independent validation of PRS’s (cohort C). Previous work on height prediction has shown that peak performance using LASSO required ~100,000 candidate SNPs derived from cohort A and a training set including >150,000 individuals from cohort B [29]. Hence, the UK Biobank cohort was divided into cohort A (50,000 individuals), cohort B (300,000 individuals) and cohort C (remaining, 44,924 individuals).

In the first step (discovery), linear mixed modeling was used on cohort A to generate p-values for association with eBMD in a manner similar to before. A p-value threshold of 3x10^-3^ was selected as there were a total of 154,990 candidate SNPs with p-values at or below this threshold, similar to the number of candidate SNPs used previously for height prediction [29].

From the set of candidate SNPs from the first step, LASSO was used to select a subset to use as predictors; specifically, Affymetrix chip type, sex, age, weight, height and ten principal components were used as covariates in the LASSO calculations using data from cohort B. Different values of the lambda penalty variable for LASSO were used, each resulting in a PRS with a different number of predictors. The range of lambda penalties was determined by testing the PRS’s for prediction of BMD on the validation set, and ensuring that the lambda values spanned the maximum performance (see below). In all, eight lambda values were tested, resulting in PRS’s consisting of between 1,306 and 131,004 predictors (Table 2). The risk score for an individual is calculated by multiplying the number of effect alleles by the effect size for each predictor, and then summing over all of the predictor SNPs.

### Prediction of ebone mineral density and osteoporosis by polygenic risk scores

Osteopenia and osteoporosis are diagnosed when BMD T scores are below -1.0 and -2.5, respectively. In the testing step, each of the polygenic risk scores was tested against BMD as well as the risk for osteoporosis. For the PRS using 1,182 SNPs with independent genome-wide significant associations with BMD (GWS_PRS), the correlation with eBMD was 0.370 (Table 2). The nine PRS’s generated using LDpred showed correlations with eBMD ranging from 0.084 to 0.305 (Table 2).

Finally, the eight PRS’s generated using LASSO were compared to eBMD for individuals in cohort C. The LASSO4 (22,886 predictors) PRS showed the highest correlation with eBMD (R = 0.415). Polygenic risk scores with either more or less predictors showed lower correlations (Table 2).

The PRS’s derived here were compared to the one used in work published previously [12]. The Estrada PRS is based on the beta values from 63 SNPs found to have a genome-wide significant association with femoral neck or lumbar spine BMD. The Estrada PRS showed a correlation of -0.143 with eBMD from cohort C, which is weaker than the correlations from the PRS developed in this work (Table 2). The Estrada risk score has a negative correlation because the beta coefficients were based on the effect for decreasing BMD, whereas the coefficients used in this study were based on the effect for increasing BMD. The lower performance of the Estrada PRS might be caused by the smaller number of SNPs used in the scoring algorithm (63 predictors versus 22,886 predictors used by LASSO4) or because the risk score from Estrada et al. was based on SNPs associated with BMD of the femoral neck and lumbar spine, rather than the heel.

**Fig 5:**
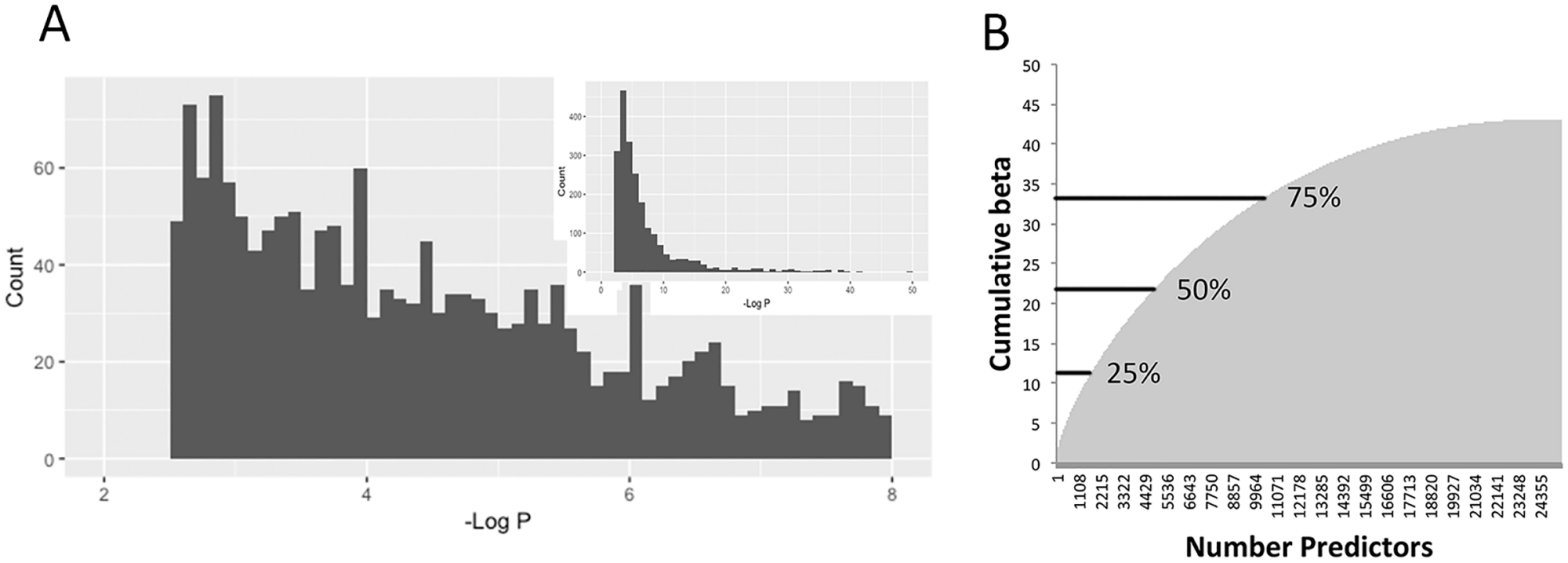
Analysis of predictors used in LASSO4. A. Histogram of p-values used by predictors in LASSO4. X-axis shows the–Log_10_ of the p-value for association with eBMD from cohort A, from p = 10^−2^ to p = 10^−8^. Inset shows data for all p-values used in LASSO4. B. Plot of cumulative score from predictors in LASSO4. X-axis is a running total of number of predictors and y-axis is the cumulative score (T-score units) from those predictors.

LASSO4 had the highest correlation with eBMD of any of the PRS’s, and hence was selected for further analysis. Fig 5A shows that the predictors used in LASSO4 contain many SNPs with p-values below genome-wide significance. This result suggests that aggregating weak predictors in the LASSO4 algorithm contributes to its performance even though some of the individual SNPs may be false positives. Fig 5B shows the cumulative contribution of each predictor to the LASSO4 algorithm. The predictors were ranked according to their effect size, and the cumulative effect was tallied. The algorithm shows a rapid rise in cumulative score from the top predictors, with 50% of the total effect contributed by the top 21% of predictors (4,885/22,886). The cumulative score plateaus at approximately 20,000 predictors, consistent with the result that adding additional predictors to the algorithm did not improve its performance.

**Fig 6:**
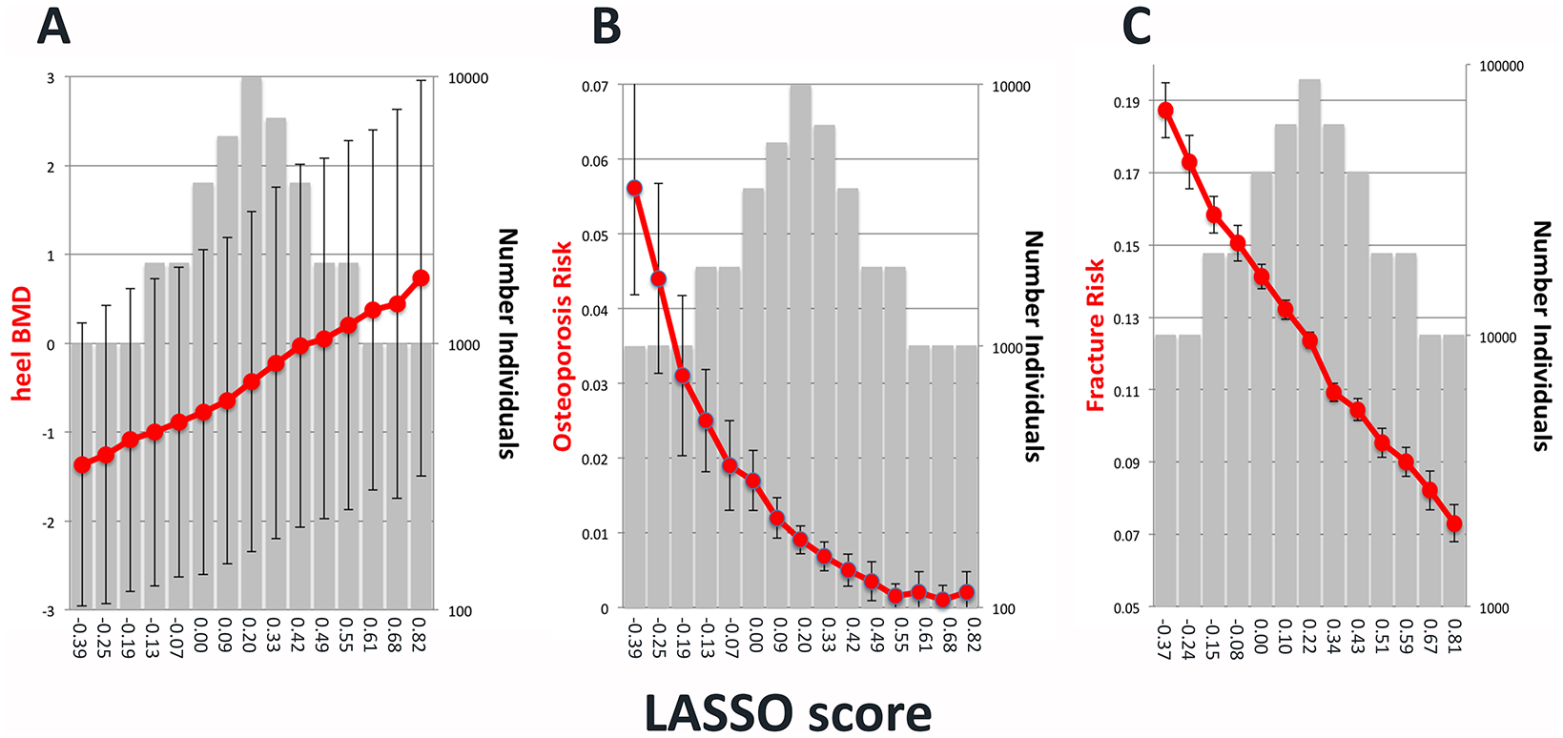
Prediction of eBMD, osteoporosis risk and fracture risk using the LASSO4 score. A. Comparison of LASSO4 with A. eBMD, B. osteoporosis risk and C. fracture risk. Individuals from cohort C were ranked based on their risk score and placed into bins. Red dots show average eBMD (T score), osteoporosis risk and fracture risk (left y-axes). Gray bars show number of individuals in each bin (right y-axis). Lowest risk score for each bin is shown on the x-axis. Error bars indicate 95% confidence intervals.

Individuals from cohort C were placed into bins according to their LASSO4 scores, and then the average eBMD was determined (Fig 6A). There was a strong upward trend for eBMD with increasing PRS. Individuals in the lowest bin (2.2% of the total) had eBMD measurements with an average T-score of -1.37 (-0.94 to 2.81; 95% CI), which was 0.934 T-score units lower than the middle bin (0. 927 to 0.942; 95% CI)(Fig 6A). Individuals in the highest bin had an average eBMD score that was 0.73 T-score units higher (-1.49 to 2.96; 95% CI) than the median.

A second approach to assess the LASSO4 PRS is to compare it to risk for osteoporosis, as osteoporosis is diagnosed when BMD T-scores are less than -2.5. There was a sharply decreasing trend for osteoporosis risk with increasing LASSO4 scores (Fig 6B). Specifically, individuals in the lowest bin for LASSO4 (2.2% of individuals) had a risk for osteoporosis that was 6.19 times higher than the middle bin (6.18 to 6.20; 95% CI). Individuals in the highest bin had a risk for osteoporosis of 0.221 (0.218 to 0.224; 95% CI) relative to the median.

Besides genetic differences, eBMD is affected by height, weight, age, sex and ancestry. To determine the effect of these factors on eBMD, a risk score using only these four covariates was generated using data from cohort B. Specifically, linear regression for eBMD was used to determine significance and assign beta values to height, weight, age, sex and the first ten principal components. The regression coefficients for height, weight, age and sex were all highly significant (p<2x10^-16^) but none of the principal components for ancestry had a significant association. A risk score using height, weight, age and sex (named Covar score) was calculated for individuals in cohort C and compared to their eBMD. The correlation between the Covar risk score and eBMD was 0.251, which is lower than the correlation between the LASSO4 PRS and eBMD (R = 0.415)(Table 2).

The LASSO4 PRS was combined with physiological covariates to determine if there was an improvement in the ability to predict ebone mineral density. Specifically, the LASSO4 score was added to Affymetrix chip type, height, age, weight, sex and 10 principal components in a linear regression for ebone mineral density, using individuals from cohort B. This showed that sex, age, height, weight and LASSO4 had significant associations with eBMD (each with p < 2x10^-16^), and determined the beta values for each covariate. The combined algorithm using LASSO4, height, weight, age and sex (termed the BOG risk score, for BMD Osteoporosis Genetic risk score) was used to calculate scores for each person in cohort C, and the scores were compared to their observed eBMD. The BOG score showed a correlation of 0.496 with eBMD, which is higher than the correlation using either the LASSO4 or Covar risk scores alone (Table 2). This result indicates that the BOG score explains about 24.6% of the variance in eBMD.

**Fig 7:**
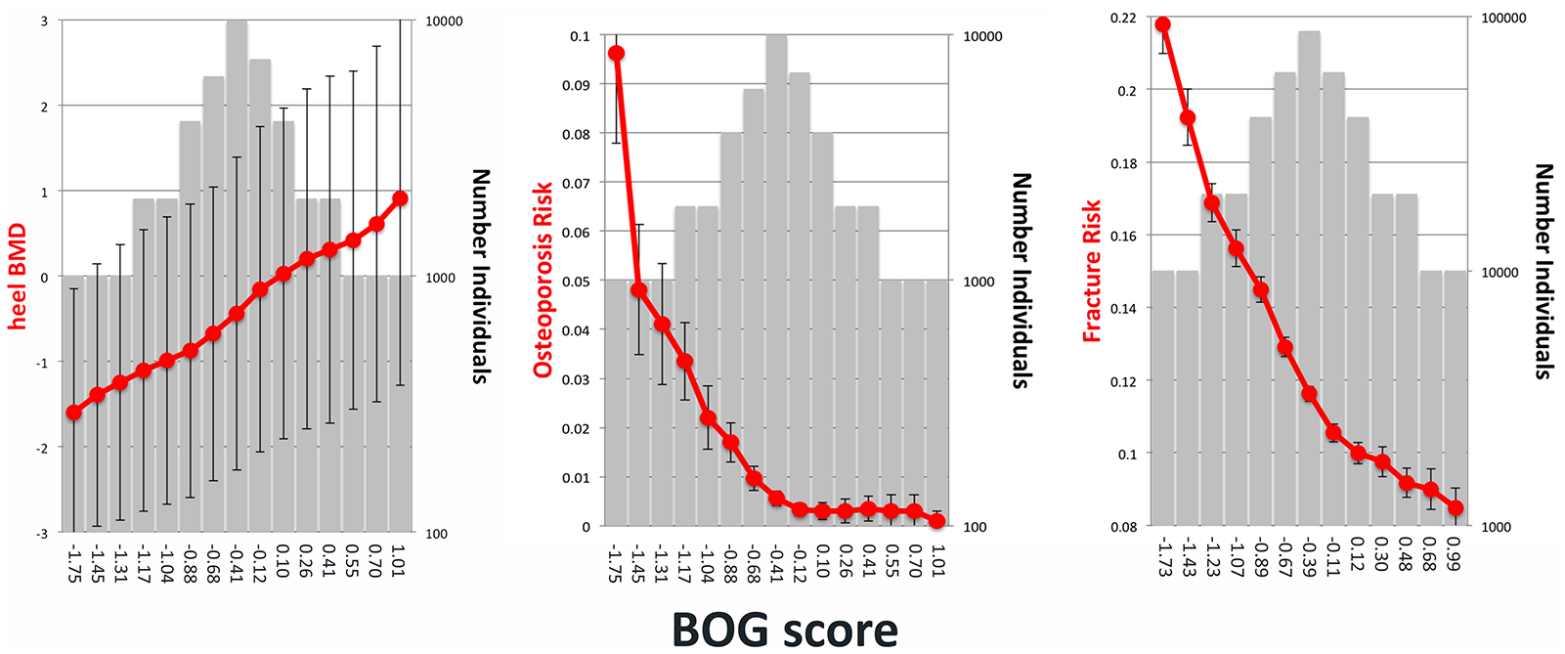
Prediction of eBMD, osteoporosis risk and fracture risk using the BOG score. A. Comparison of the BOG risk score with A. eBMD, B. osteoporosis risk and C. fracture risk. Individuals from cohort C were ranked based on their risk score and placed into bins. Red dots show average eBMD (T score), osteoporosis risk and fracture risk (left y-axes). Gray bars show number of individuals in each bin (right y-axis). Risk score for each bin is shown on the x-axis. Error bars indicate 95% confidence intervals.

Individuals from cohort C were placed into bins according to their BOG scores, and each bin was compared to eBMD and risk for osteoporosis (Fig 7A and 7B). Individuals in the lowest bin (2.2% of total) had an average eBMD measurement of -1.60 T-score units (-3.05 to 0.15; 95% CI), which is 1.16 T-score units lower than the middle bin (1.15 to 1.17; 95% CI). The lowest bin had a risk for osteoporosis that was 17.37 times higher than the middle bin (17.35 to 17.38; 95% CI).

### Prediction of risk for fractures by genetic risk scores

Since low bone mineral density is a risk factor for bone fracture [26,30,31], the risk scores for eBMD were compared to incidence of fracture. There were 59,378 participants in the UK Biobank that had bone fracture of any type; the cases of fracture were identified by self-reported responses to a questionnaire (47,494 individuals) or by inpatient electronic health records (23,686 individuals)(S6 Table). The incidence of fracture data was not used to either select SNPs or to train the genetic risk scores, so the genetic risk scores were tested on the entire genotyped UK Biobank population.

Using the LASSO4 and BOG algorithms, the score for each genotyped individual in the UK Biobank cohort was calculated. For LASSO4, individuals in the lowest bin had a 1.52 fold increased risk for bone fracture relative to the middle bin (1.51–1.53; 95% CI)(Fig 6C). Individuals in the highest LASSO4 bin had a relative risk of fracture of 0.590 (0.584–0.596; 95% CI). For BOG, individuals in the lowest bin had a 1.88 fold increased risk for bone fracture relative to the middle bin (1.87 to 1.88; 95% CI)(Fig 7C).

**Fig 8:**
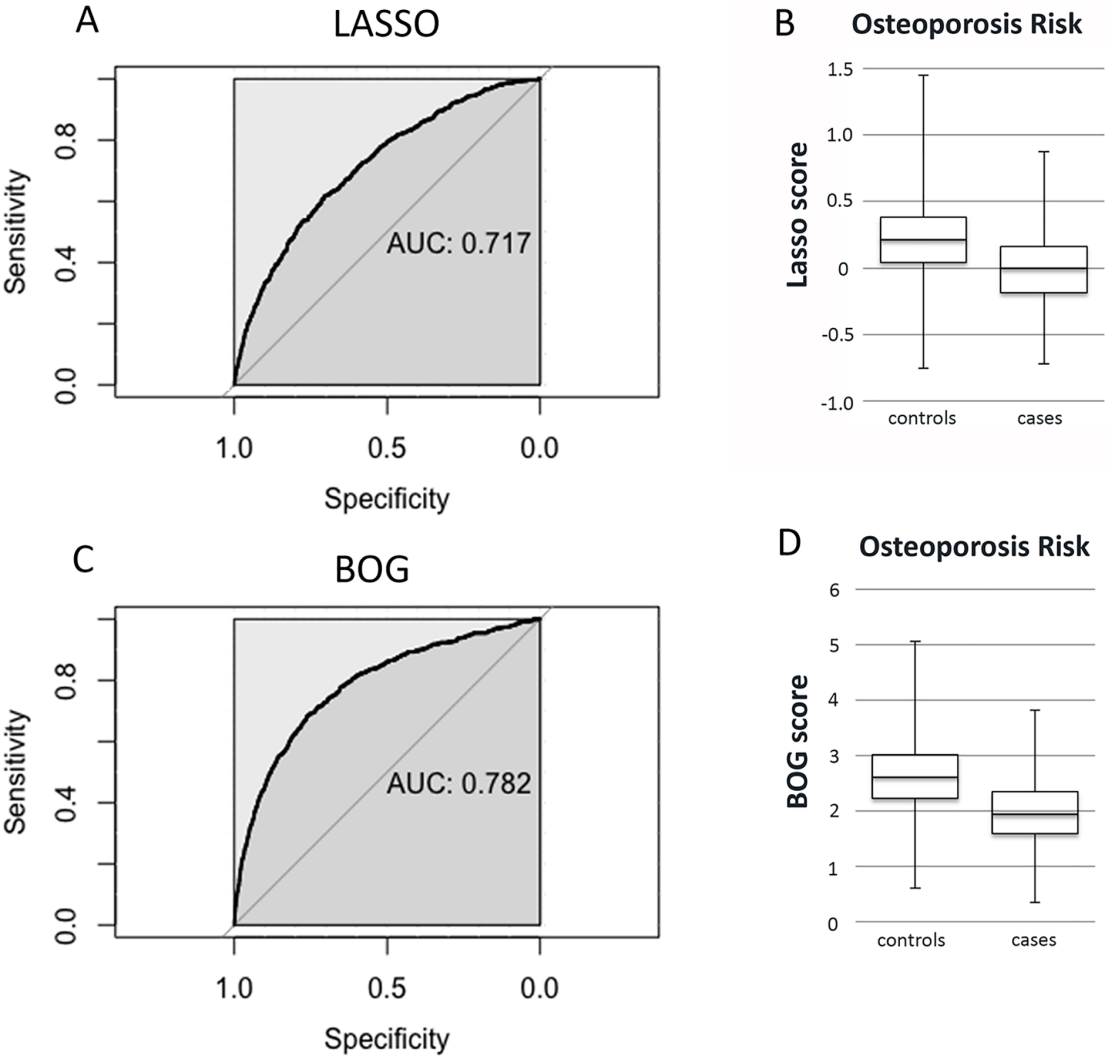
Receiver Operator and box plots for PRS’s for osteoporosis risk. A. Receiver Operator plot for the LASSO4 PRS for osteoporosis. B. Box plot for the LASSO 4 PRS. C. Receiver Operator curve for the BOG score for osteoporosis. D. Box plot for the BOG score. AUC; Area Under the Curve. Box plots show the maximum, first quartile, mean, third quartile and minimum scores for LASSO4 and BOG for osteoporosis cases and controls.

Although the BOG risk algorithm has a robust ability to identify a subset of individuals with higher risk of osteoporosis and fracture, its overall ability to discriminate cases from controls is modest. For instance, the first BOG bin has a risk of osteoporosis and fracture of 9.6% and 21.8%, indicating that most are unaffected (i.e. false negatives). Fig 8 shows Receiver Operator curves and box plots for LASSO and BOG scores for osteoporosis. For BOG, the area-under-the-curve for osteoporosis and fracture are 0.782 and 0.570, respectively (S7 Table). By comparison, the area-under-the-curve for fracture using eBMD as a predictor is 0.600. The box plots show that the BOG scores are higher for osteoporosis than controls. The discrimination slope is the difference in mean BOG values between controls and cases; for osteoporosis and fractures, the discrimination slope is -0.674 and -0.149, respectively (S7 Table). The discrimination slopes show that there is a statistically significant decrease in BOG score in cases compared to controls for osteoporosis and fractures.

## Discussion

A genetic association study was performed for eBMD using the full release of data from UK Biobank including 394,929 individuals. A total of 1,362 independent SNPs were identified with a genome-wide significant association to eBMD. These SNPs clustered into 899 loci, of which 286 were previously identified and 613 are new. Consistent with previous results, this study shows that part of the genetic basis for the eBMD phenotype is polygenic, with many common variants contributing small effects [12,13,15]. Sequencing results have previously shown that eBMD is also influenced by rare variants, such as rs11692564 near the EN1 gene [32].

A high percentage of previously-identified SNPs were validated using the data from this study. Specifically, Maoyyeri et al. and Mullin et al. collectively identified a total of 22 SNPs with a genome-wide association to eBMD, and all were strongly validated in this study [13,14]. Estrada et al. performed a genetic association study for BMD of the lumbar spine and femoral neck using dual-energy x-ray absorptiometry (DXA) [12]. Of the 62 genome-wide significant SNPs that were identified, 57 were validated in this study. The SNPs that were not validated might be false positives, might reflect differences in bone structure measured by DXA versus QUS, or might be specific for different anatomical sites (spine or hip vs the heel). Kemp et al. identified SNPs with a genome-wide specific association with eBMD using UK Biobank data that partially overlapped the data from this study, so the analyses are not independent [15]. Nevertheless, 304 of the 307 SNPs identified by Kemp et al. showed a significant association to eBMD in this study. This observation shows that nearly all of the original associations were maintained following the addition of new data.

The BOG risk score was developed to predict levels of eBMD, risk of osteoporosis and risk of fracture. This algorithm includes genotype information from 22,886 DNA predictors along with four physiological parameters (height, weight, age and sex). The correlation with eBMD for the BOG algorithm was 0.496, which is higher than the correlations achieved using the 22,886 predictors (R = 0.415) or the four covariates (R = 0.251) alone. This result emphasizes the importance of combining DNA information with physiological factors to predict risk for low BMD or injury. The correlation of the BOG score with eBMD was also higher than the correlation using 1,182 SNPs from this study (R = 0.370) or 63 SNPs from a prior study (R = -0.143) that have genome-wide significant associations with BMD [12]. The BOG algorithm explains about 24.6% of the variance in eBMD levels. By comparison, the algorithm developed by Estrada et al. explained about 2.0% of the variance in BMD levels in this study, and 5.8% of the variance in elderly women from a previous study [12].

Individuals with low BOG scores were found to have lower eBMD levels (-1.16 T-score units) than the median BOG score. The identification of a group of individuals with low average eBMD may be clinically important as osteopenia is diagnosed with T-scores ranging from -1.0 to -2.5 and osteoporosis is diagnosed with T-scores below -2.5. Specifically, individuals with low BOG scores had a 17.4 fold increased risk for osteoporosis compared to the median BOG score. Finally, low BOG scores were associated with 1.88 fold higher risk for bone fractures than the median BOG values. Using the algorithm developed by Estrada et al. and tested on elderly women, individuals with the highest risk score had a decrease in BMD levels of 0.33 units, an increase in risk for osteoporosis of 1.56 fold and an increase in risk for fracture of 1.60 fold, compared to the median risk score [12].

Although the BOG algorithm identifies rare individuals at risk for low BMD, osteoporosis and fracture (i.e. those in the lowest bin), its ability to discriminate cases from controls in the overall population is modest. Using osteoporosis as an example, about 90% of individuals with BOG scores in bin 1 (the high risk bin) are unaffected (false negatives) and about 0.1 to 0.5% of individuals in bins 8–15 (lower risk bins) have osteoporosis (false positives). The area-under-the-curve for predicting osteoporosis or fracture by the BOG algorithm is 0.78 and 0.57, respectively.

BMD defines the cutoff for osteoporosis and is the strongest risk factor for fracture [33,34]. Having a prior fracture is also a strong risk factor for having an additional fracture [35,36]. These two factors and 7 additional clinical risk factors have been incorporated into the FRAX index, which is widely used to predict risk of fracture [34,37,38]. The genetic markers identified in this study have greatest utility when BMD, prior fracture history and FRAX score are not known. Genome-wide genotype information is becoming routinely available for large numbers of people due to various precision medicine initiatives. In contrast to genotype information, BMD and FRAX assessment are not routinely measured for the general population. Hence, an algorithm such as the BOG risk score might be useful to screen the general population in order to identify individuals that warrant closer examination, such as BMD measurement via DXA.

The BOG risk score could be used to predict injury similar to the way that clinical genetics is used to predict disease. With clinical genetics, individuals carrying a risk allele at the disease locus are rare, but the effect of the risk allele is large. For instance, mutations in the *BRCA2* gene occur in about 0.2% of women and result in about a seven-fold increase in the lifetime risk of breast cancer [39]. Likewise, with the BOG algorithm, individuals with a low risk score are rare (2.2%), but the effect of the score on risk for low BMD, osteoporosis and fracture can be substantial (e.g. 17-fold increase of osteoporosis). Using the BOG score developed here, the cumulative effects of 22,886 SNPs are summed, enabling the identification of rare individuals with a high number of risk alleles. The effect of each individual SNP is inconsequential but the cumulative effect from all of the SNPs can be large. In this fashion, the BOG risk score converts common SNPs with weak effects to a rare genetic score with a large effect.

One limitation is that this study only included participants of European ancestry regardless of their level of physical activity. The results of this study should be validated for other ancestry groups. The results should also be validated in a cohort where all are physically active, such as athletes and military personnel. Physical activity has a large effect on BMD and fracture. Weight-bearing exercises increase one’s bone mineral density, but also increase one’s chance of bone fractures (due to falls or collisions), including stress fractures due to repetitive training. Athletes and military personnel have much higher risks of stress fractures than the overall UK Biobank cohort due to the harsh demands of physical training [3,4].

The genetic association analysis for BMD was limited to the heel. The effects of the SNPs identified in this study on BMD for other anatomical sites (such as the lumbar spine or femoral neck) are unclear. From a previous study of genetic associations with the femoral neck and lumbar spine BMD, most but not all of the SNPs were associated with BMD at diverse sites, but a few were specific for BMD for either the lumbar spine or femoral neck [12].

Widespread genetic testing using the BOG risk score could identify individuals with increased risk for low BMD, osteoporosis or fracture. This could identify individuals that should undergo further testing, especially tests to directly measure BMD. For elderly women, this could identify individuals with osteoporosis, thereby enabling steps to be taken to prevent fragility fractures before they occur. For athletes and military personnel, genetic testing could be used to identify those at risk for bone fractures, including stress fractures. For elite athletes, bone/stress fractures can interrupt training, cause important competitions to be missed and can thus be a disadvantage against competitors. For elite military personnel, bone/stress fractures can reduce military effectiveness, prevent completion of training, and increase the overall cost of training. Hence, the impact of bone/stress fractures on competitive success for athletes and physical performance for military personnel can have significant consequences beyond their impact on health. Genetic testing could inform athletes and military personnel about their risk of injury, enabling preventative diet, exercise and training regimens to be used before injury occurs.

## Methods

### Phenotypes

Data on eBMD for 394,929 individuals were obtained from UK Biobank (URLs). Calcaneal quantitative ultrasound data (QUS) were obtained from UK Biobank, as described in Kemp et al. [15]. Heel bone mineral density (eBMD) was estimated based on an ultrasound measurement of the calcaneus by UK Biobank. The T-score is the number of standard deviations for bone mineral density relative to the mean. Consistent with the criteria established by Kemp et al., individuals were excluded that exceeded the following thresholds for eBMD: males, ≤0.18 or ≥1.06 g/cm2; females ≤0.12 or ≥1.025 g/cm2 [15]. Further details on these methods are publicly available on the UK Biobank website (URLs).

The gold standard for diagnosing osteoporosis is to measure BMD with dual-energy X-ray absorptiometry (DXA). QUS gives results comparable to DXA and can also be used as a pre-screening tool for cases of osteoporosis validated by DXA. In this study, osteoporosis cases were identified as participants with BMD T-scores below -2.5 from cohort C using QUS. There were 455 cases of osteoporosis and 28,819 controls.

Incidence of fracture was obtained from two sources. The first was from a questionnaire regarding self-reported incidence of fracture in the past five years. Individuals were excluded if they responded “Do not know” or “Prefer not to answer”. There were 47,494 individuals with fracture and 451,457 controls identified from the questionnaire. The second source was from in-patient electronic health records based on fracture diagnoses using the 10th revision of the International Statistical Classification of Diseases and Related Health Problems (ICD-10) code. The list of codes and number of individuals diagnosed with that code are shown in S5 Table. There were 23,686 individuals identified by their electronic health records. In total, there were 59,378 participants in the UK Biobank that had any type of bone fracture.

Data on height, weight, age and sex were obtained from UK Biobank (URLs). Height, weight and age refer to values at the time of enlistment. Age was calculated from the date of enlistment and the participant’s birthday. Sex was determined by genotype.

### Genotypes

Genotype data for 488,378 participants were obtained from the v3 release of UK Biobank [18]. The genotypes of the UK Biobank participants were assayed using either of two genotyping arrays, the Affymetrix UK BiLEVE Axiom or Affymetrix UK Biobank Axiom array. Genotype data were imputed centrally by UK Biobank with IMPUTE2 using the Haplotype Reference Consortium and the UK10k+ 1000GP3 reference panels [40]. Metrics for quality control were established and then used to filter DNA variants by UK Biobank [18]. HLA genotypes were determined by UK Biobank [18]. Bycroft et al. describe the full details regarding genotyping of the UK Biobank participants [18]. Variants were excluded that failed UK Biobank quality control procedures in any of the genotyping batches, that showed a departure from Hardy-Weinberg of P<10^−50^, or that had a MAF < .001. The resulting list contained 20,259,828 genotyped and imputed DNA variants.

Individuals were excluded who were identified by the UK Biobank as outliers based on either genotyping missingness rate or heterogeneity, whose sex inferred from the genotypes did not match their self-reported sex, who withdrew from participation or who were not of European ancestry.

### GWAS analysis

To search for genetic association with eBMD while taking into account population structure in the UK Biobank cohort (e.g. presence of related individuals), LMM-BOLT was used to perform a Linear Mixed Model [19]. Specifically, the model takes the form

y=XB+g+E

where y is the vector of eBMD, X is the matrix of fixed effects, and β is the effect size of these fixed effects. Fixed effects included sex, array batch, age, height, weight, and the leading 10 genomic principal components as computed by UK Biobank. g is the polygenic effect that captures the population structure. The Genomic Relationship Matrix (GRM) was computed using common (MAF > 0.001) genotyped variants that passed quality control. E is a residual effect not accounted for by the fixed and random effects. To account for the large number of SNPs in the UK Biobank release, a threshold of 6.6x10^-9^ was used for genome-wide significance, as established by Kemp et al. [15]. Upon acceptance, summary statistics for all SNPs from the linear mixed model will be made available at NIH GRASP: https://grasp.nhlbi.nih.gov/FullResults.aspx.

Genetic association with bone fracture was assessed by linear mixed modeling using LMM-BOLT [19]. The fixed effects were the same as those used for eBMD. The Genomic Relationship Matrix was computed similarly to the GRM for eBMD.

Linear regression was used to search genotyped DNA variants from the pseudo-autosomal region of the Y chromosome, mitochondrial DNA variants and HLA loci for associations with eBMD. Co-variates included sex, array batch, age, height, weight, and the leading 10 genomic principal components as computed by UK Biobank. To prevent errors caused by relatedness among the individuals in the UK Biobank cohort, the analysis was restricted to unrelated individuals, defined as those that were used to define the principal component values by UK Biobank [18]. Linear regression was performed with PLINK2.0 [20].

Quantile-quantile (Q-Q) and Manhattan plots were generated in R using the qqman package [41]. The lambda genomic control and LD score regression intercept were calculated using LMM-BOLT [19]. Locus plots were created using LocusZoom [42]. The chromosomal locations and annotation of SNPs were determined using Annovar [22]. The effects of missense SNPs on protein function were analyzed using SIFT and POLYPHEN2 [43,44]. Missense SNPs were included as potential causal variants if the log of their p-value was within 50% of the log of the p-value for the sentinel SNP. Regulatory SNPs were identified using data from ENCODE [24], as reported by RegulomeDB [23].

DNA variants showing an independent genome-wide-significant association with eBMD were identified using GCTA COJO [21]. They were then assigned to a chromosomal locus, defined as a region where the gap between independent genome-wide significant DNA variants was less than 100 kb.

### Polygenic risk scores for eBMD, osteoporosis and bone fracture

LDpred was used to calculate the posterior mean effect size for each variant based on summary p-values from linear mixed modeling and subsequent shrinkage based on the extent to which the variant was correlated with similarly associated variants in the UK Biobank population [19]. The summary statistics for association with ebone mineral density were obtained using linear mixed modeling on 350,000 individuals. SNPs with p-values less than 0.1 were selected for analysis in order to reduce computational memory requirements. The linkage disequilibrium data were obtained from 50,000 European individuals. LDpred includes a variable indicating the fraction of markers that have non-zero effect sizes. The default values included in the LDpred package were used: 1, 0.3, 0.1, 0.03, 0.01, 0.003, 0.001, .0003 and .0001. The LDpred package also removes SNPs with ambiguous strand or minor allele frequency less than 1%. The resulting LDpred risk scores included effect sizes for 1,497,447 SNPs.

PLINK1.9 was used to perform the least absolute shrinkage and selection operator (LASSO) [20]. LASSO requires an estimate of heritability (h2) as input, which was calculated using BOLT-REML from cohort A [19]. Different values of the penalty parameter, lambda, were used resulting in different numbers of predictors. The genetic algorithms contain a set of predictors with the effect allele and a beta coefficient for the effect allele. Score is calculated by weighting each effect allele by its beta coefficient, and then summing over all effect alleles. Effect alleles associated with an increase in BMD have positive beta coefficients, and vice versa when the effect allele has a negative association.

In the testing step, the genetic risk algorithms were used to calculate scores for individuals in cohort C. The correlation between the genetic risk scores and observed eBMD was calculated using R.

LASSO4 was selected for further analysis to determine whether the algorithm could be strengthened by combining it with height, weight, age and sex. The beta values for these factors were used to generate a combined risk score termed the BOG (BMD Osteoporosis Genetic) risk score. Individuals were placed into bins based on the risk scores. The 95% confidence interval was calculated using the modified Wald method. The area-under-the-curve for each of the risk scores was calculated using the pROC package in R (URLs).

### URLs

UK Biobank (UKBB), http://www.ukbiobank.ac.uk.laneproxy.stanford.edu/; UK Biobank protocol for measurement of eBMD, https://biobank.ctsu.ox.ac.uk/crystal/docs/Ultrasoundbonedensitometry.pdf; UK Biobank document #155580 on genotyping and quality control, http://biobank.ctsu.ox.ac.uk/crystal/docs/genotyping_qc.pdf; Haplotype Reference Consortium, http://www.haplotype-reference-consortium.org/site; pROC package, http://web.expasy.org/pROC/.

## Supporting information

S1 Table Summary statistics for 142,417 SNPs with genome-wide significant associations with eBMD. (XLSX)

S2 Table Results from conditional analysis for association with eBMD. (TXT)

S3 Table Missense SNPs that have p-values the same or similar to the sentinel SNP for their loci. (XLSX)

S4 Table Regulatory SNPs for eBMD. (TXT)

S5 Table Validation of previously-identified SNPs with associations to BMD. (XLSX)

S6 Table ICD-10 codes for fractures from in-patient electronic health records. (DOCX)

S7 Table AUC and discrimination slopes for the polygenic risk algorithms for risk for osteoporosis and fracture. (DOCX)
