# Correction: Identification of 613 new loci associated with heel bone mineral density and a polygenic risk score for bone mineral density, osteoporosis and fracture

**DOI:** 10.1371/journal.pone.0213962

**Published:** 2019-03-13

**Authors:** Stuart K. Kim

In the “Identification of DNA variants associated with eBMD” subsection of the Results, there is an error in the fourth sentence of the second paragraph. The linkage disequilibrium score regression intercept is incorrectly written as 1.21. The correct intercept score is 1.05. This value indicates that the association analysis is not confounded by population structure 1.

The author would like to provide additional comments regarding the limitations of the study. First, osteoporosis is defined based on heel ultrasound measurements rather than DXA-derived bone mineral density. Second, all fractures included in the study are prevalent fractures based on data either from a self-reported questionnaire or from electronic health records. Third, risk factors (height, age, weight and sex) are measured at the time of recruitment into the study rather than prior to injury. These limitations are acknowledged in the Methods section of the manuscript.
