# Supplementary material for: Identification of 613 new loci associated with heel bone mineral density and a polygenic risk score for bone mineral density, osteoporosis and fracture
Source: PLoS One. 2018 Jul 26;13(7):e0200785. doi: 10.1371/journal.pone.0200785 (PMC6062019; doi:10.1371/journal.pone.0200785)
Supplement: S6 Table — (DOCX) [file pone.0200785.s006.docx]

**S5 Table. ICD-10 codes for fractures from in-patient electronic health records.**

| ICD-10^a^ | Description | Number^b^ |
| --- | --- | --- |
| S02 | Fracture of skull and facial bones | 2,484 |
| S12 | Fracture of neck | 375 |
| S22 | Fracture of rib(s), sternum and thoracic spine | 2,235 |
| S32 | Fracture of lumbar spine and pelvis | 1,605 |
| S42 | Fracture of shoulder and upper arm | 3,106 |
| S52 | Fracture of forearm | 7,436 |
| S62 | Fracture at wrist and hand level | 7,522 |
| S72 | Fracture of femur | 2,674 |
| S82 | Fracture of lower leg, including ankle | 6,683 |
| S92 | Fracture of foot, except ankle | 1,250 |

^a^ 10th revision of the International Statistical Classification of Diseases and Related Health Problems code.

^b^ Number of individuals with in-patient records containing this ICD-10 code.
