# Supplementary material for: Identification of 613 new loci associated with heel bone mineral density and a polygenic risk score for bone mineral density, osteoporosis and fracture
Source: PLoS One. 2018 Jul 26;13(7):e0200785. doi: 10.1371/journal.pone.0200785 (PMC6062019; doi:10.1371/journal.pone.0200785)
Supplement: S7 Table — (DOCX) [file pone.0200785.s007.docx]

**S6 Table. AUC and discrimination slopes for polygenic risk algorithms for risk for osteoporosis and fracture.**

| Name | AUC Osteo (95% CI)^a^ | Disc. Slope Osteo (95% CI)^b^ | AUC Fracture (95% CI)^c^ | Disc. Slope Fracture (95% CI)^d^ |
| --- | --- | --- | --- | --- |
| Lasso4 | 0.717 (0.695 to 0.738) | -0.217 (-0.135 to -0.299) | 0.562  (0.557 to 0.568) | -0.054  (-0.057 to -0.052) |
| GWS_GRS | 0.604 (0.590 to 0.615) | -0.130 (-0.106 to -0.154) | 0.543  (0.536 to 0.549) | -0.058  (-0.050 to -0.065) |
| Estrada_GRS | 0.553 (0.526-0.580) | -0.035  (-.025 to -.045) | 0.530 (0.523 to 0.542) | -.0189  (-0.153 to -0.225) |
| Covar^f^ | 0.664  (0.634 to0.694) | 0.002  (-.017 to 0.020) | 0.533 (0.524 to 0.543) | 0.033  (0.026 to 0.040) |
| BOG^g^ | 0.782  (0.761 to 0.803) | -0.674 (-0.623 to -0.724) | 0.570  (0.565 to 0.576) | -0.149. (-0.147 to -0.152) |

^a^ Area_under_the_curve for risk for osteoporosis.

^b^ Discrimination slope for osteoporosis is the difference in mean between cases and controls.

^c^ Area_under_the_curve for risk for fracture.

^d^ Discrimination slope for fracture is the difference in mean between cases and controls.
